# Lateral Surgical Approach for Subungual Glomus Tumors With Low Recurrence Rates: A Single-Center Case Series

**DOI:** 10.7759/cureus.85869

**Published:** 2025-06-12

**Authors:** Roberto Martínez-Mejorada, Gabriel García-González, Alejandro Curiel-Rivas, David A de la Garza-Kalife, Rodolfo Franco Márquez, Melissa F Ochoa-Cortez, Yanko Castro-Govea, Mauricio M García-Pérez

**Affiliations:** 1 Plastic and Reconstructive Surgery, Hospital Universitario Dr. José Eleuterio González, Universidad Autónoma de Nuevo León, Monterrey, MEX; 2 Human Anatomy, Hospital Universitario Dr. José Eleuterio González, Universidad Autónoma de Nuevo León, Monterrey, MEX; 3 General Surgery, Hospital Universitario Dr. José Eleuterio González, Universidad Autónoma de Nuevo León, Monterrey, MEX; 4 Biochemistry and Molecular Medicine, School of Medicine, Universidad Autónoma de Nuevo León, Monterrey, MEX

**Keywords:** case series, fingertip, glomus tumor, hand, mesenchymal neoplasm, mexico, plastic surgery, surgical excision, transungual approach

## Abstract

Introduction: Glomus tumors are rare benign mesenchymal neoplasms typically presenting as small, painful, blue-red nodules with cold sensitivity. Surgical excision with complete removal of the tumor capsule is the standard treatment for benign glomus tumors. Recurrence is uncommon but can occur if the tumor is not fully excised.

Methods: This retrospective, single-center case series includes a sample of 24 patients with glomus tumors treated surgically between 2017 and 2023 by the Department of Plastic and Reconstructive Surgery at the Hospital Universitario Dr. José Eleuterio González, Monterrey, Mexico.

Results: Glomus tumors were more common in women (n=18, 75%), and affected the left hand in 17 cases (70.8%) and the subungual region in 11 cases (45.8%). The thumb and index digits were the most frequently affected, while the third and fifth were the least. The average duration of symptoms before diagnosis was 2.9 years. A single case had a history of recurrence, in which the participant had been previously treated at another facility before we performed the second resection.

Conclusion: A lateral surgical approach with careful matriectomy was effective in most cases, with minimal recurrence. The case with previous recurrence underscores the importance of complete excision.

## Introduction

Glomus tumors are rare mesenchymal neoplasms composed of modified smooth muscle cells that arise from the glomus body, a contractile neuromyoarterial apparatus that functions as a thermoregulator through arteriovenous shunting of blood [1-4]. Although frequently found in the subungual regions of digits or the deep dermis of the palm, wrist, forearm, and foot due to their high concentrations of glomus bodies, glomus tumors may occur anywhere, including lung, stomach, pancreas, liver, gastrointestinal, and genitourinary tract [1-5]. Out of all glomus tumors, 65% occur on the fingertips, and most of these have a subungual location, with only a few found in the volar pulp of the finger [6]. They usually form a bulging encapsulated mass with an irregular, nodular, blue-red surface [1,2]. These tumors may be subcategorized as solid glomus tumor, glomangioma, or glomangiomyoma based on their glomus cell, vasculature, and smooth muscle cell composition [1-3]. Solid glomus tumor, the variant with predominantly glomus cells, represents 75% of all glomus tumors [1,3,7]. Even though their potential to become malignant glomangiosarcomas is exceedingly rare, with very few reports in the literature [2,3], benign glomus tumors may be significantly disabling due to their intense, localized pain and sensitivity to cold. In some cases, they may even progress to bone invasion, underscoring the importance of recognizing their clinical presentation.

Patients typically present with blue-red nodules, smaller than 1 cm, associated with localized tenderness, cold sensitivity, and excruciating paroxysmal pain out of proportion to the neoplasm size [1,4-6,8]. Under normal conditions, cold temperatures cause relaxation of the glomus body and open the arteriovenous anastomosis, diverting blood away from the capillary network to conserve body heat [9]. Hypotheses state that glomus tumors can cause pain elicited by temperature changes or tactile stimulation, probably because of reflex vasodilation that precipitates a temperature change of the entire extremity [1,8]. Although previous studies showed that nerve fibers and cyclooxygenase-2 [8], as well as mast cells releasing heparin, histamine, and 5-hydroxytryptamine, are abundant in glomus tumors [10], the mechanisms behind this painful presentation are not fully understood. This lack of understanding, the rarity of glomus tumors, and their often small, non-palpable presentation may explain why they usually remain undiagnosed for several years (on average 3.3-5 years) with patients seeing an average of 2.5 physicians (range: 0-7) before the diagnosis of a glomus tumor is confirmed [5].

While the clinical presentation and typical subungual location may be highly suggestive of a glomus tumor, definitive diagnosis requires histologic assessment by biopsy or excision [11]. Immunohistochemical analysis confirms the diagnosis by revealing glomus cells that, regardless of their epithelioid features and close association with vasculature, are positive for smooth muscle actin, muscle-specific actin, calponin, h-caldesmon, vimentin, and collagen type IV; while negative for cytokeratin and S100 [1,2]. Imaging with possible diagnostic use includes radiography, ultrasonography, and magnetic resonance imaging (MRI), but this is not usually necessary since diagnosis is based on clinical presentation along with tests such as Love’s pin test, Hildreth’s test, cold sensitivity test, and a transillumination test [10,12,13]. Differential diagnoses to keep in mind when seeing a patient with severe pain in the tip of the finger include nodular hidradenoma, dermal nevi, myopericitoma, angioleiomyoma, eccrine spiradenoma, hemangioma, neuroma, osteochondroma, or mucous cyst [1,10]. Fortunately, once a glomus tumor is diagnosed correctly, the treatment has a good prognosis.

Benign glomus tumors are treated effectively by surgical excision with complete removal of the tumor capsule [3,6,9,14]. Direct transungual excision, which consists of cutting through the nail bed, is the standard of treatment because it offers a good field of view [6,9,10]. Nevertheless, this surgical approach can cause postoperative nail deformity; the lateral subperiosteal approach for tumors partially under the nail reduces the risk of unsatisfactory cosmetic results but may slightly raise the risk of recurrence [15,16]. Lateral approaches can be subperiosteal, in which an incision is made dorsally to the mid-lateral line and a dorsal flap is raised containing the skin, nail bed, and germinal matrix, or lateroungual (Keyser-Littler), in which the interosseous ligament of the distal phalanx is identified and retracted, lifting the matrix over the ligament and periosteum of the distal phalanx [7]. Another approach for peripheral subungual tumors, while inadequate for central visualization, is the modified periungual approach, an L-shaped incision over the periungual area that reveals the tumor without needing nail bed repair [10]. These disadvantages can be offset with techniques such as guiding incision by anatomic location, using a tourniquet to enhance visualization of the tumor, dissecting layer by layer in skin-colored tumors to include the whole capsule, and assisting the surgical excision with a microscope to remove the glomus tumor completely while minimizing damage to the nail bed [6,10,12]. In cases where the tumor is apposed to the bone, it may be necessary to perform bony curettage to prevent the recurrence of symptoms [9]. Recurrence is low (10%) and only found in cases of incomplete excision or missed diagnosis of coexisting small glomus tumor [3,6,14].

Although case series of glomus tumors are well published and widely concordant in their management, there is currently no description of cases in the northern region of Mexico. Thus, this descriptive study of 24 cases of glomus tumors treated between 2017 and 2023 by the Department of Plastic and Reconstructive Surgery at the Hospital Universitario Dr. José Eleuterio González was performed. An evaluation of the clinical, diagnostic, and intraoperative findings in patients with glomus tumors was done to provide epidemiologic value to glomus tumor management in northern Mexico, which can aid in profiling regionally specific clinical presentation and guide management replication.

## Materials and methods

This was a retrospective, single-center, formal case series with clinical-based sampling. The study was performed at the Department of Plastic and Reconstructive Surgery, Hospital Universitario Dr. José Eleuterio González, Monterrey, Mexico, covering cases from 2017 to 2023. The study was approved by the Research Ethics Committee of the Hospital Universitario Dr. José Eleuterio González (approval number: CP23-00008). Patient information was de-identified using anonymized data protocols to protect confidentiality.

Only patients with confirmed glomus tumors who underwent surgical intervention were included, while those with incomplete medical records were excluded. Recruitment was carried out through a medical records search.

The therapeutic intervention consisted of surgical excision of the tumor, conducted by attendings and residents from the Department of Plastic and Reconstructive Surgery, all of whom had relevant training and specialization in managing glomus tumors. Measures to ensure quality control included adherence to a standard surgical technique to minimize variations. The participants were reached by telephone and asked about recurrence symptoms. No participants were lost to follow-up throughout the study period, ensuring comprehensive data collection and outcome assessment. This case series has been reported in line with the PROCESS (Preferred Reporting Of CasE Series in Surgery) Guideline [17].

## Results

A total of 32 cases presented over the five years of the study period, and due to incomplete files or follow-up, only 24 cases of glomus tumors were analyzed (Table 1). There were 18 women (75%) and six men (25%), with an average age of 42.2 years. The incidence of glomus tumor by anatomical location was seven cases (29.2%) on the right hand and 17 cases (70.8%) on the left hand. Twenty patients (83.3%) reported the affection on their dominant hand; five cases (20.8%) on the thumb, six cases (25%) on the index finger, two cases (8.3%) on the middle finger, four cases (16.6%) on the ring finger, two cases (8.3%) on the small finger, and five cases (20.8%) on other locations of the hand. Only one case of recurrence was found, in which the patient had been previously treated in a different center before we performed the second resection.

**Table 1:** Demographic profile of case series participants

Patient	Age (years)	Sex	Finger	Laterality	Subungual	Color	Time (months)	Pain	Size (mm)
1	36	F	1	Left	Yes	Violet	180	Mild	10 x 10 x 3
2	23	F	4	Right	Yes	Light brown	72	Severe	4 x 3 x 2
3	31	M	2	Left	Yes	Light brown	3	Severe	12 x 8 x 4
4	33	F	2	Left	Yes	Light brown	24	Severe	6 x 5 x 2
5	50	F	-	Right	No	Light brown	7	Moderate	3 x 4 x 2
6	35	F	1	Left	Yes	Light brown	12	Moderate	8 x 6 x 3
7	55	M	-	Right	No	Pink	72	Severe	4 x 3 x 3
8	59	F	1	Left	Yes	Violet	24	Severe	6 x 4 x 2
9	41	M	4	Left	Yes	Light brown	24	Severe	7 x 3 x 3
10	58	F	2	Left	No	Yellow	60	Moderate	5 x 4 x 3
11	28	F	5	Left	Yes	Light brown	180	Severe	10 x 10 x 3
12	39	M	-	Left	No	Red	36	Severe	5 x 4 x 4
13	47	F	-	Left	No	Brown	7	Moderate	12 x 3 x 2
14	55	F	2	Left	No	Red	24	Severe	4 x 3 x 4
15	73	F	3	Left	No	Brown	13	Moderate	11 x 6 x 6
16	38	F	3	Right	No	Blue	9	Moderate	20 x 20 x 10
17	57	M	4	Left	No	Red	18	Moderate	25 x 10 x 5
18	52	F	2	Left	No	Brown	10	Mild	3 x 4 x 2
19	7	F	4	Left	No	Brown	5	Moderate	6 x 2 x 3
20	26	F	1	Left	Yes	Violet	20	Moderate	13 x 10 x 8
21	49	F	5	Left	No	Brown	6	Moderate	4 x 2 x 2
22	30	F	1	Right	No	Red	24	Moderate	5 x 5 x 2
23	51	F	-	Right	Yes	Light brown	6	Moderate	4 x 4 x 2
24	42	M	2	Right	Yes	Brown	14	Moderate	4 x 3 x 2

The patients reported pain in the distal region of the affected finger, erythematous pigmentation in the nail bed, localized tenderness, and temperature sensitivity. We used a lateral approach in all cases, except one, which was managed with a transungual approach and horizontal block resection. In cases with radiographic findings of bone lysis, bony curettage was performed. The surgical approach used in most patients began with a lateral incision of the nail edge, after which the nail was lifted, revealing the macroscopic characteristics of the tumor (usually a localized growth of the nail bed), and matricectomy was performed by dissecting the sub-matrix plane. Then, the tumor was excised, the nail matrix and skin flap were sutured, and a nail splint was placed, ending the surgical procedure. All cases of surgical excision were sent for histopathological study. Two cases from this study are described below to illustrate our findings.

Case A

**Figure 1:**
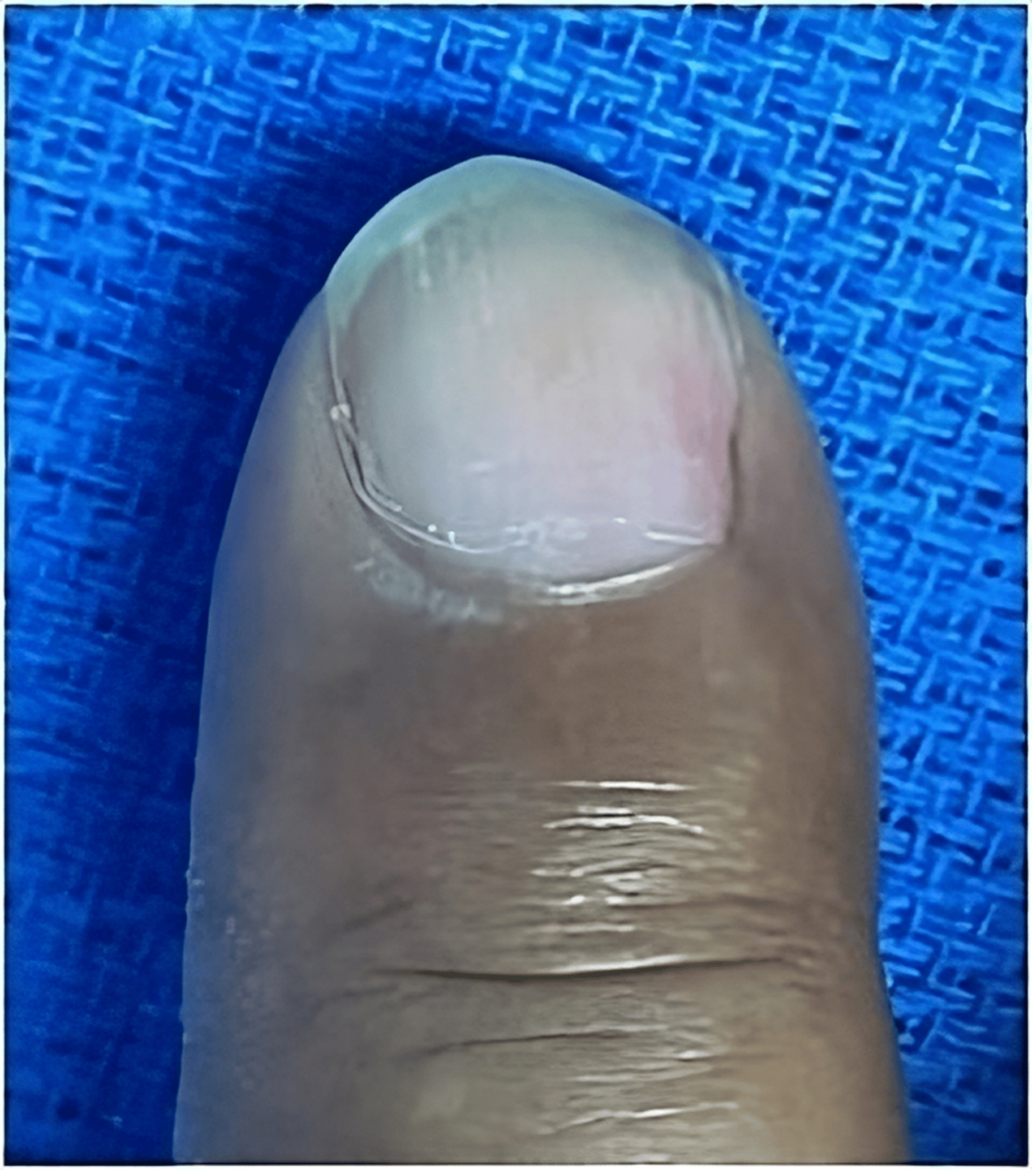
Clinical presentation in Case A Macroscopic presentation of subungual glomus tumor seen as a reddish spot.

A 42-year-old man, a writer by profession, presented to the Department of Plastic Surgery with a nail deformity. He had no history of chronic diseases, only a contusion of the distal phalanx of the right index finger two years prior, which subsequently developed into localized, severe pain exacerbated by pressure and cold temperature, with reddish pigmentation on the nail edge (Figure 1), for which a doctor diagnosed onychocryptosis and performed a nail resection. Despite the procedure, the pain persisted and increased progressively, for which the patient went to Algology and received anesthetic infiltrations and analgesics without noticeable improvement.

**Figure 2:**
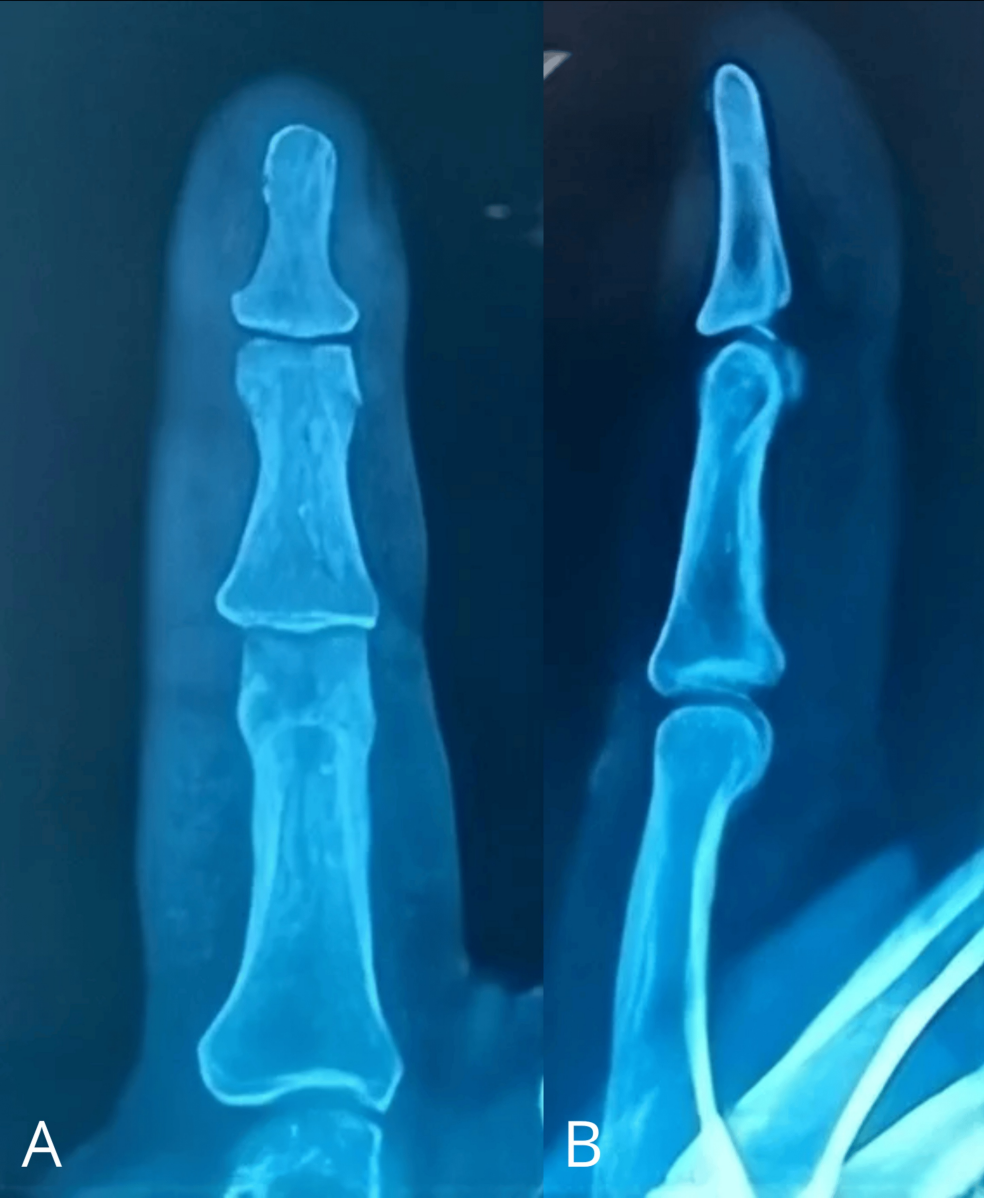
Radiographic examination of Case A Anteroposterior (A) and lateral (B) views of the right second digit.

He was then referred to the Department of Plastic and Reconstructive Surgery of the Hospital Universitario Dr. José Eleuterio González for evaluation. On examination, a clinically reddish spot was observed, with sharp punctiform pain and nail deformity of elevation at the paronychial level (Figure 1). No evidence of cortical lysis or other synchronous lesions was found on plain radiographic examination (Figure 2).

**Figure 3:**
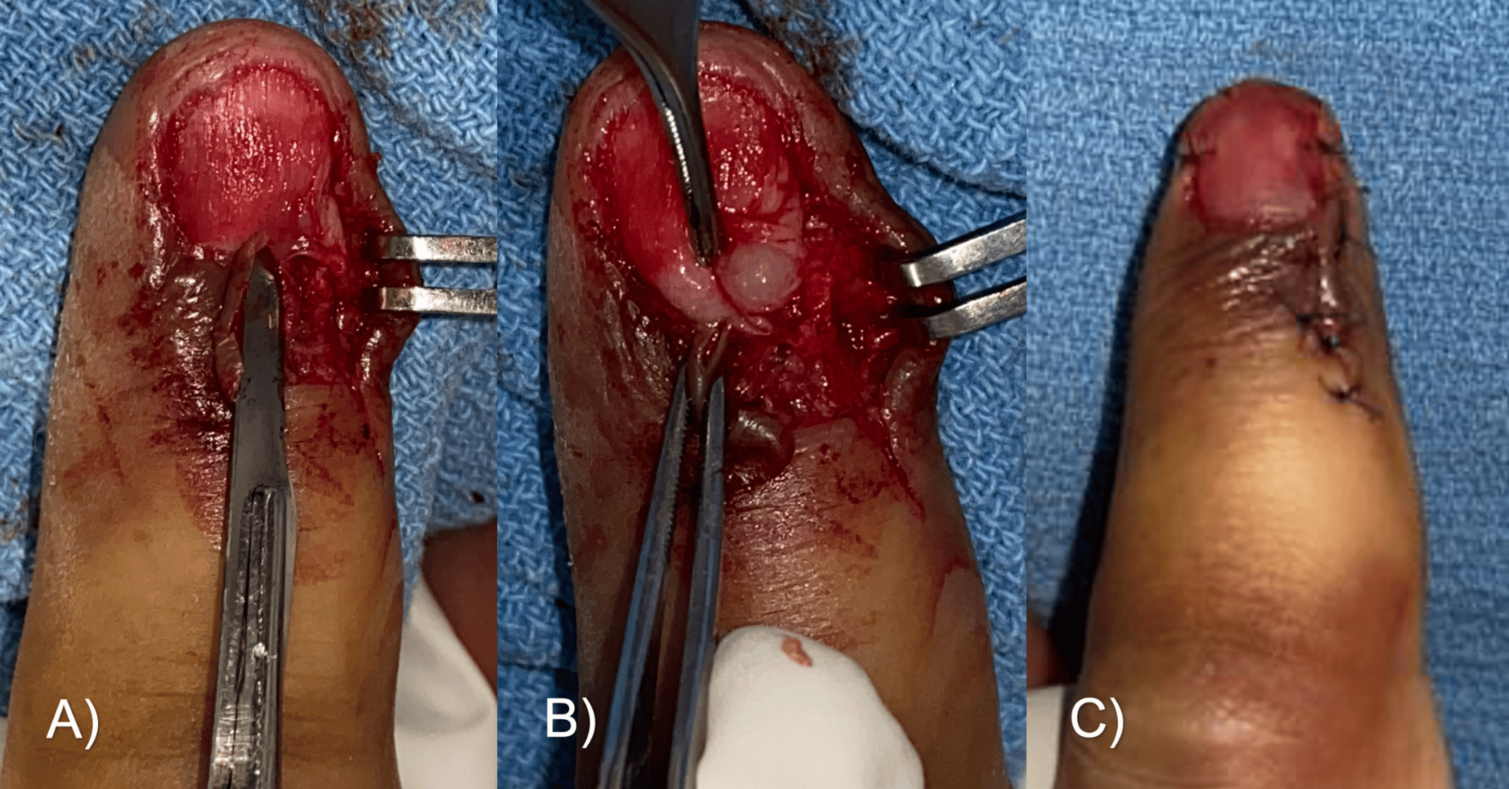
Surgical removal of the glomus tumor in Case A (A) An approach was made at the base of the nail, on the lateral side of the dorsum of the finger, and the nail was removed to expose the nail bed. (B) Tumor dissection. (C) Suturing and nail fixation.

**Figure 4:**
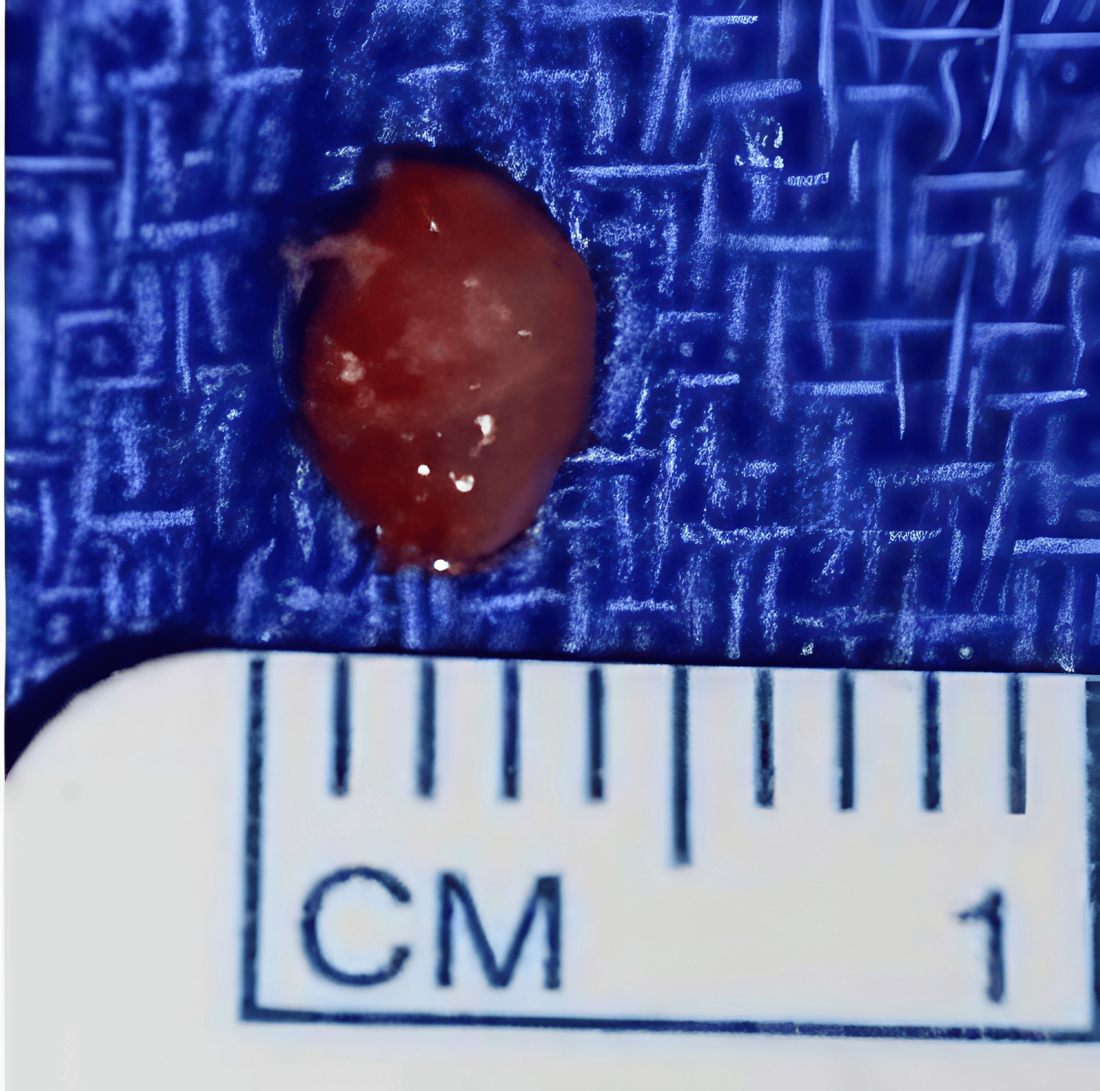
Resected glomus tumor in Case A Glomus tumor, 3 x 4 mm, with brown and ovoid appearance.

We decided to use a lateral approach to excise the glomus tumor. In the surgical procedure, a lateral incision was made to the nail edge and the nail was lifted to observe an increase in volume at the site of the lesion (Figure 3). A lateral matricectomy was performed, dissecting in the sub-matriceal plane, to find a tumor of 3 x 4 mm, ovoid in shape, and brown, which was resected along with three hypertrophic corpuscles (Figure 4). Bony curettage was not performed. The nail matrix was sutured with polyglycolic acid 6-0, a skin flap, and an autologous nail splint was placed.

**Figure 5:**
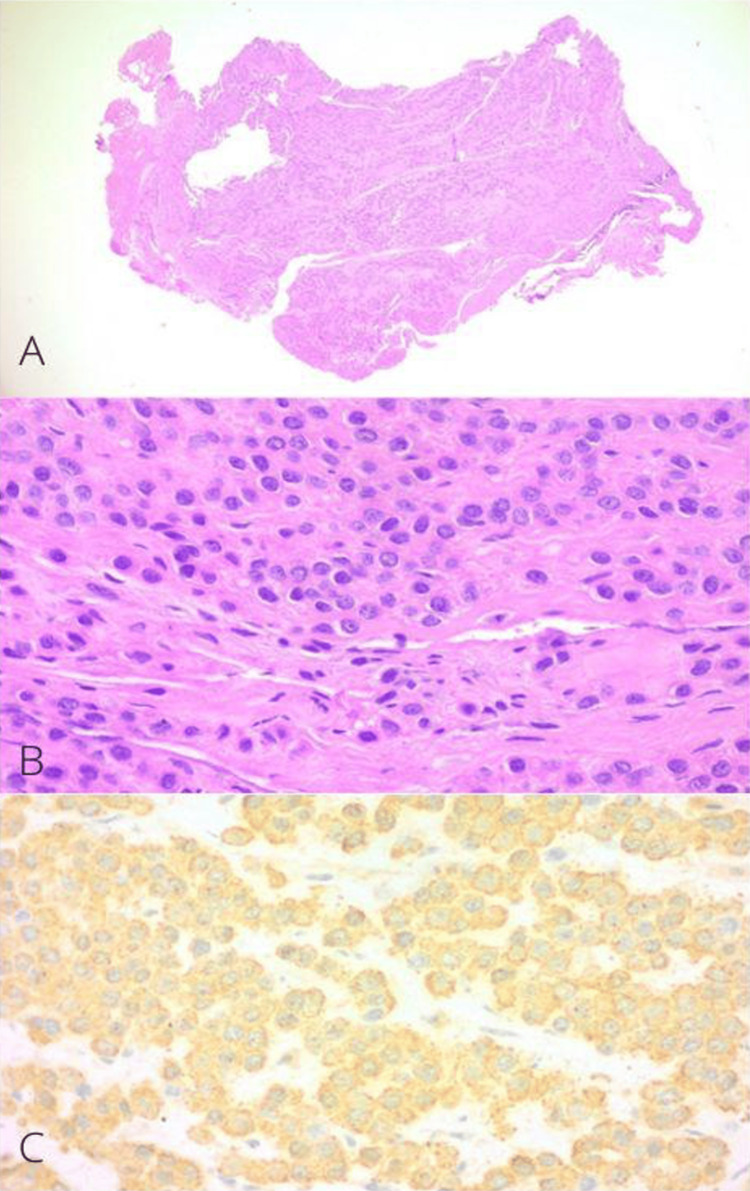
Histopathology of glomus tumor in Case A (A) Panoramic histologic section of a lesion composed of soft cell sheets, round cells, and some capillaries (H&E, 10x). (B) Glomus cells surrounded by capillaries (H&E, 40x). (C) Positivity for immunohistochemistry "smooth muscle actin" (40x).

**Figure 6:**
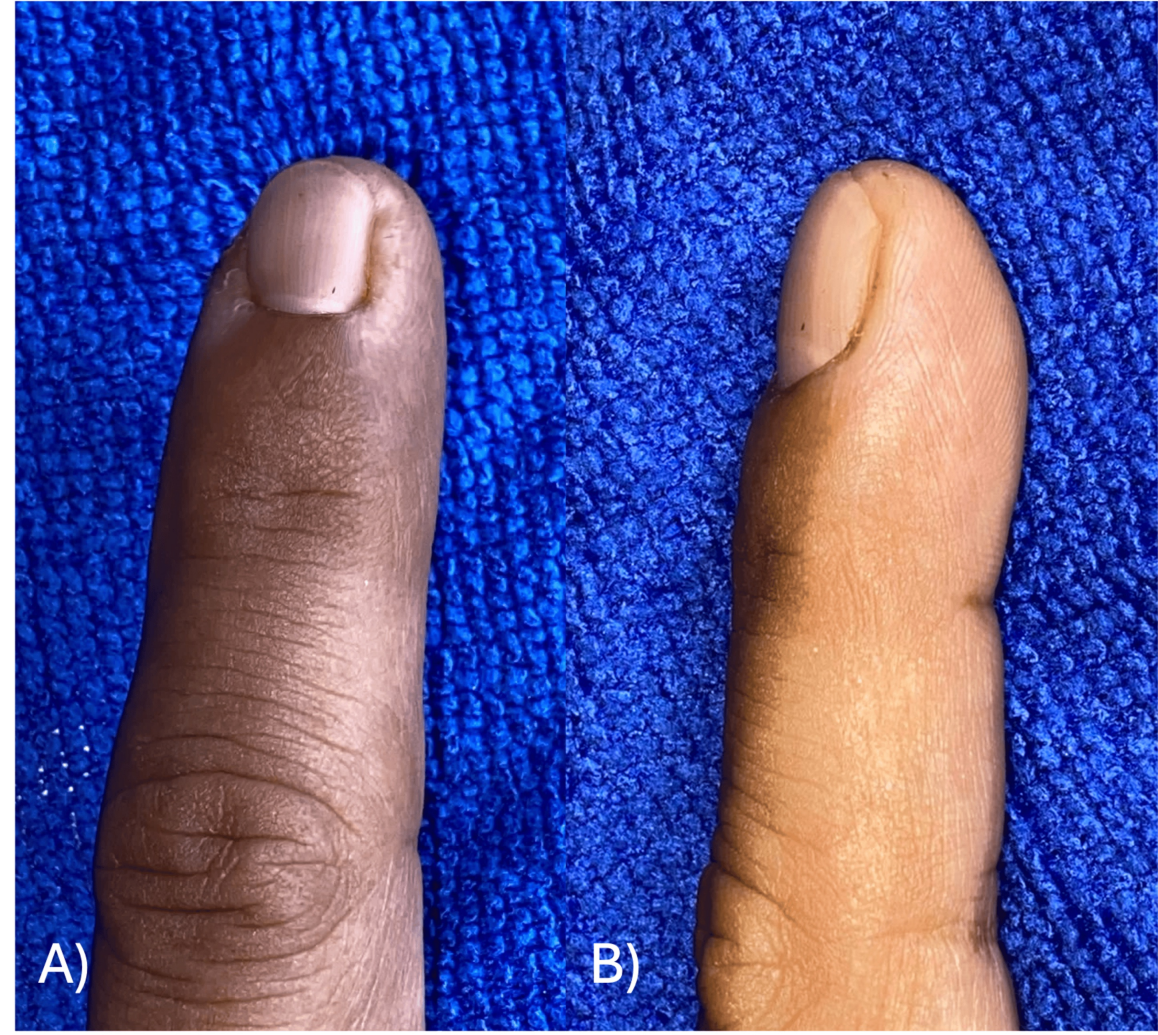
Images from the one-year follow-up in Case A There was no pain and very satisfying functional and aesthetic results.

The tumor was sent for histopathology, which reported a glomus tumor with positive immunohistochemistry for smooth muscle actin (Figure 5). In the patient’s follow-up, the nail splint was removed after four weeks. The wound healed adequately, and there was no recurrence of symptoms at the one-year follow-up (Figure 6).

Case B

**Figure 7:**
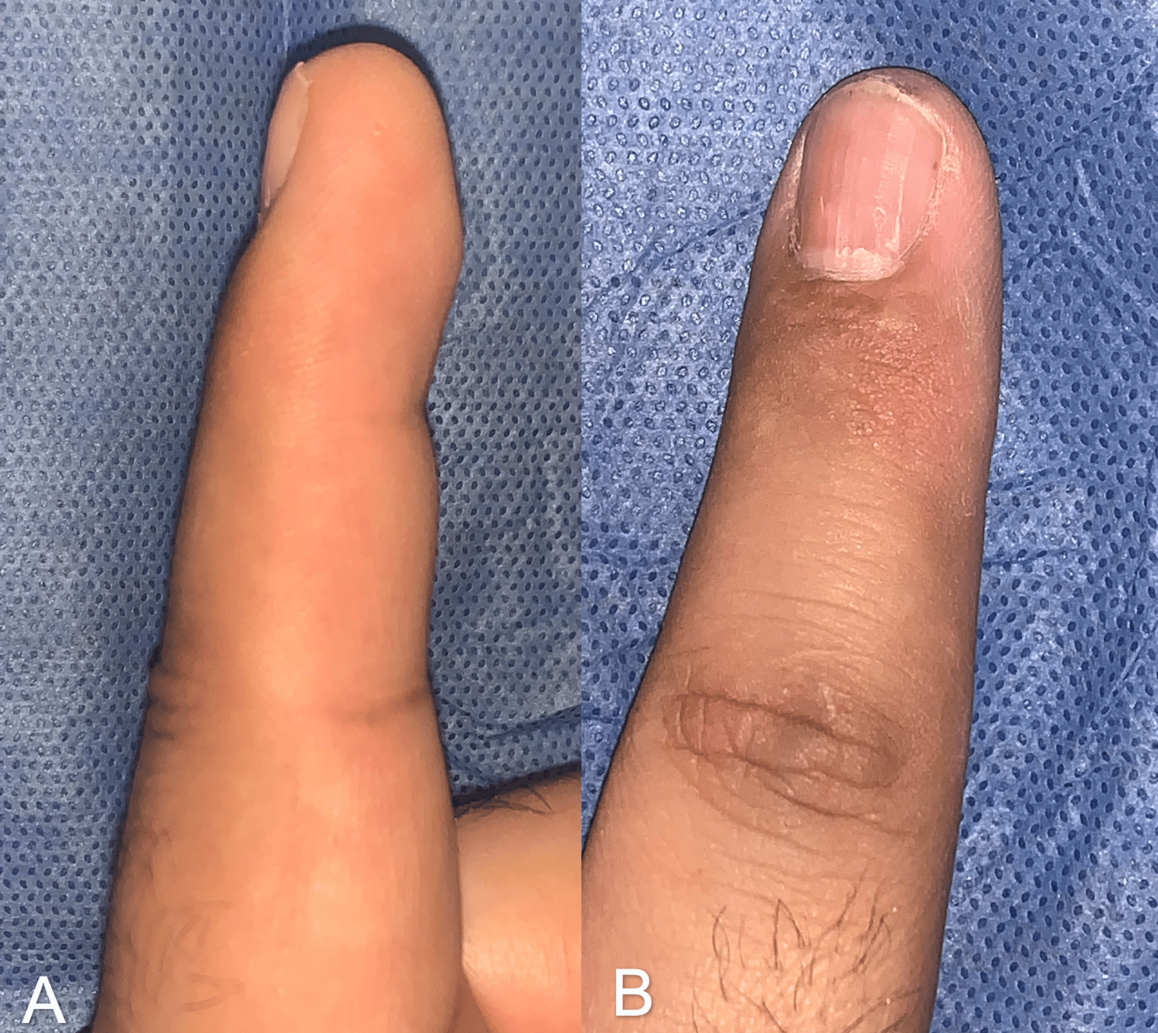
Lateral (A) and anteroposterior (B) view of the subungual glomus tumor with no nail deformities in Case B

**Figure 8:**
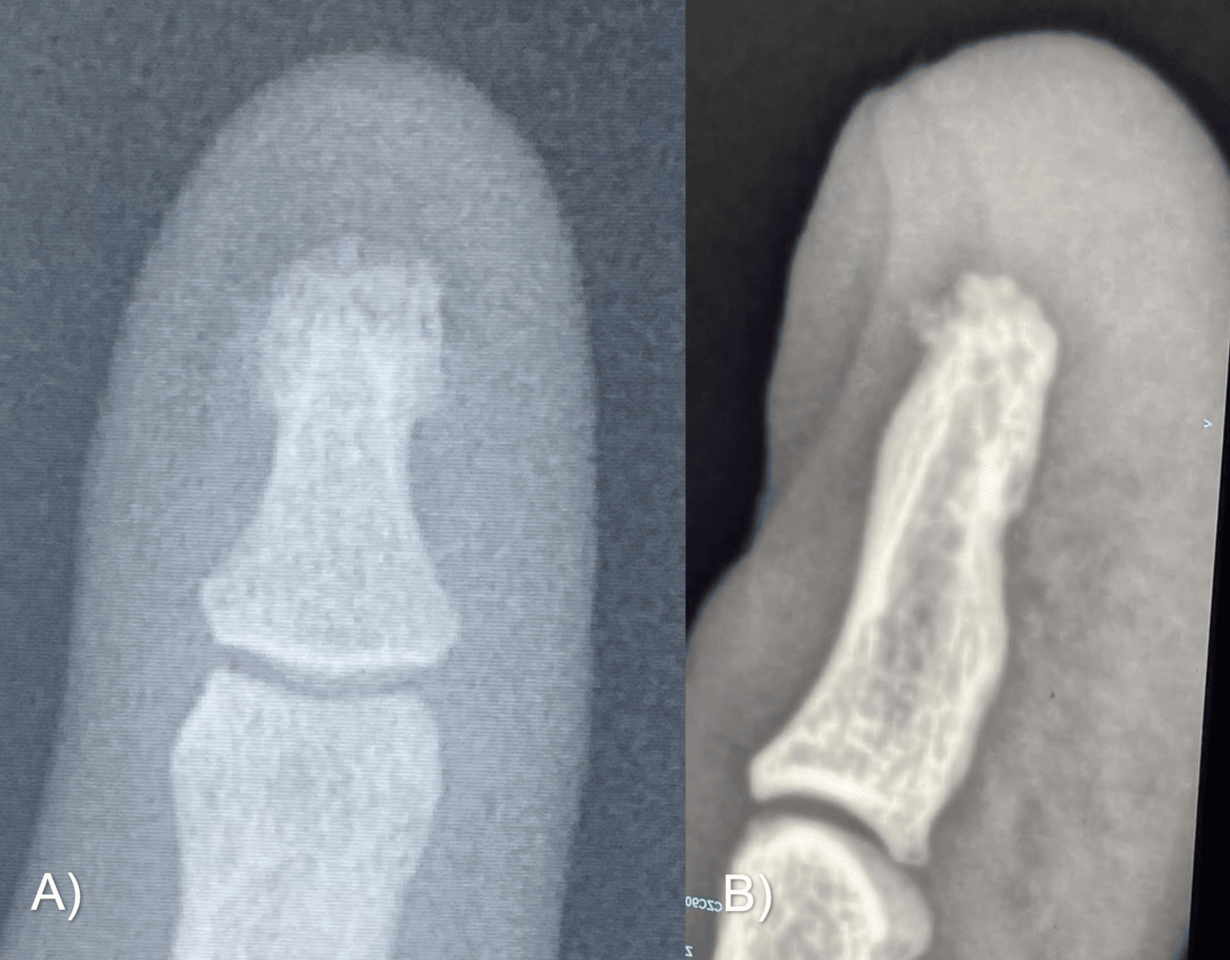
Radiographic examination in Case B Anteroposterior (A) and lateral (B) views of the left second digit with cortical lysis in the tuft of the distal phalanx

A 31-year-old man, a diver by profession, presented with gradually increasing, localized pain in his left index finger, which worsened with pressure and cold temperature. He had no history of chronic diseases, only a previous glomus tumor in his left index finger, which was resected and confirmed by histopathology five years before the current referral consultation. No nail deformities were found on physical examination, only intense pain in the hyponychium below the free edge of the nail (Figure 7). Plain radiographs showed evidence of cortical lysis in the tuft of the distal phalanx (Figure 8).

**Figure 9:**
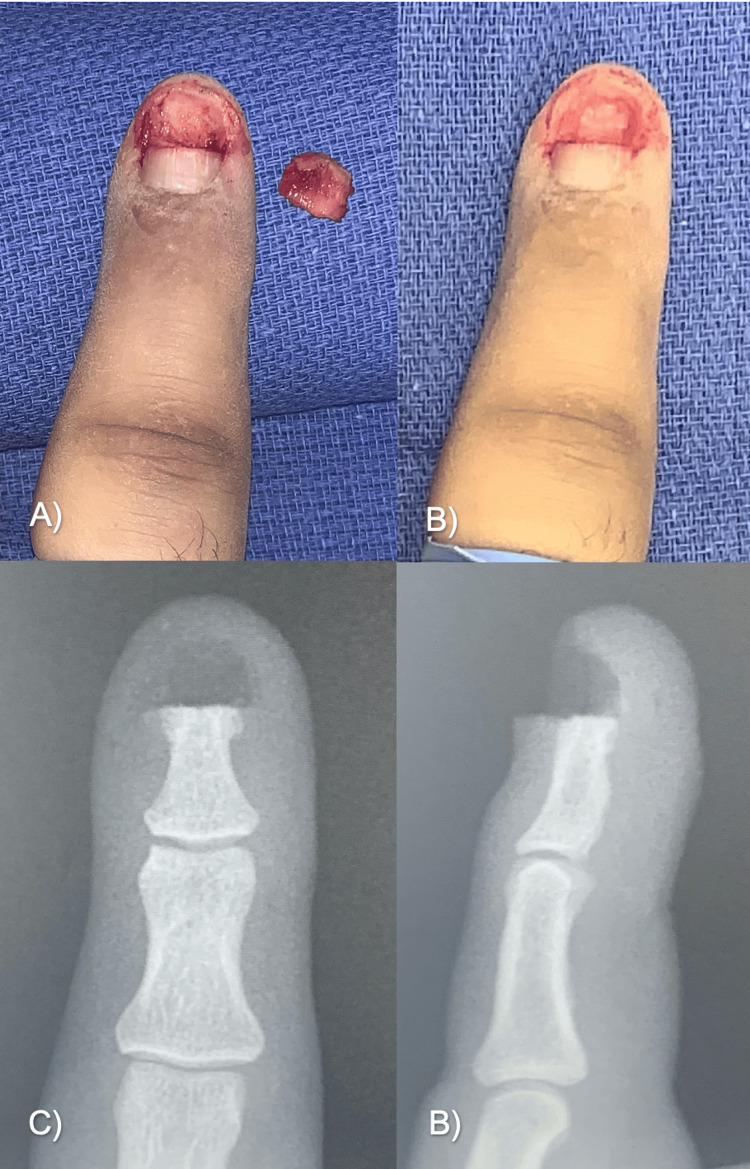
Surgical procedure matricectomy through a transungual approach in Case B Resection (A,B). Evidence of bony curettage at the site of the glomus tumor seen from anteroposterior (C) and lateral (D) views.

**Figure 10:**
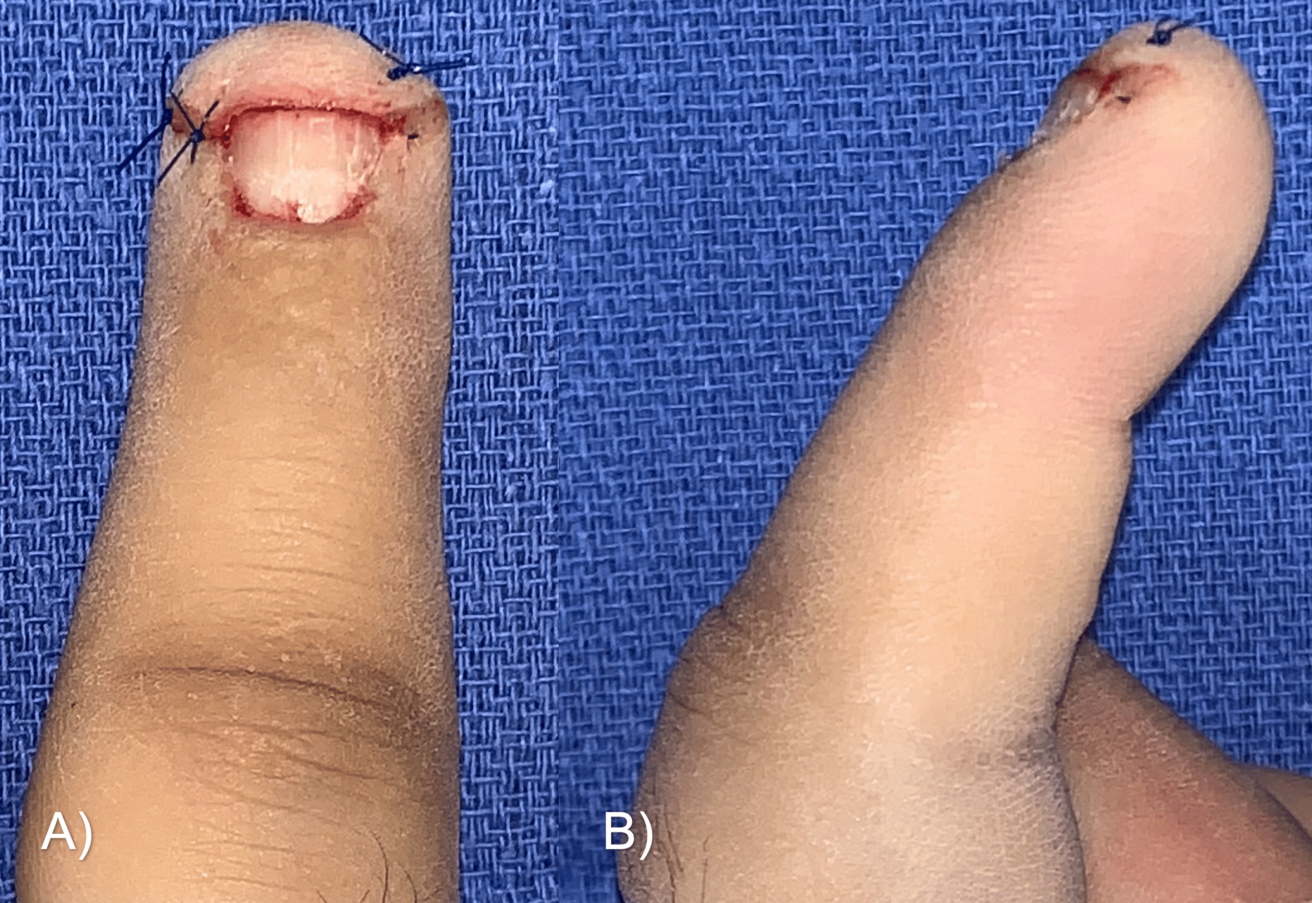
Postoperative result in Case B Cutaneous-adipose flap of half of the distal phalanx to cover the remaining nail seen from anteroposterior (A) and lateral (B) views.

Nail excision was performed following a transungual approach, which begins with a transverse incision at the junction of the middle and distal thirds of the nail (Figure 9). The cut was deepened down to the tuft of the phalanx, then the most distal area was resected along with the nail matrix using a gouge, resulting in a cutaneous-adipose flap of half of the distal phalanx. This was slightly thinned and approximated with a filled flap to cover the remaining nail, and the lateral redundant skin folds were managed with triangular incisions and finally sutured (Figure 10). Onychoplasty was performed to improve the nail height appearance.

**Figure 11:**
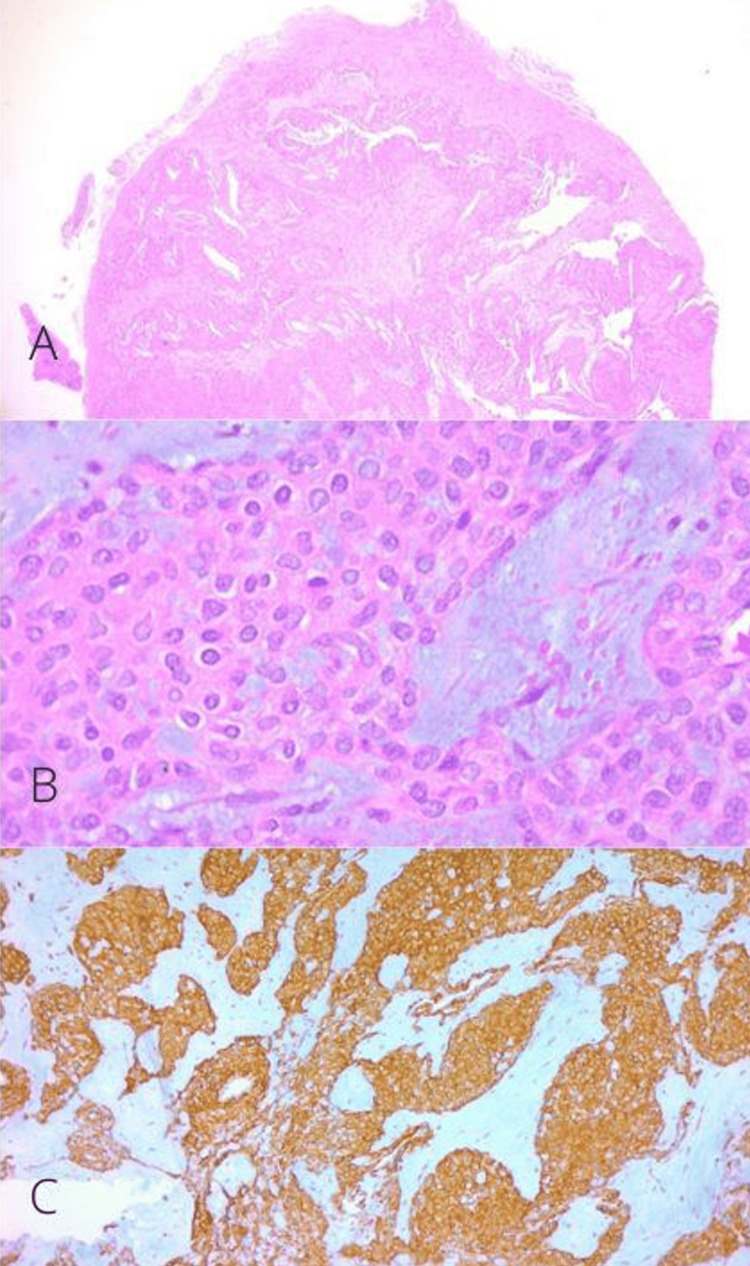
Histopathology of glomus tumor in Case B (A) Histologic section of the well-circumscribed lesion (H&E, 10x). (B) Glomus cells surrounded by capillaries within a myxoid stroma (H&E, 40x). (C) Positivity for immunohistochemistry "smooth muscle actin" (40x).

**Figure 12:**
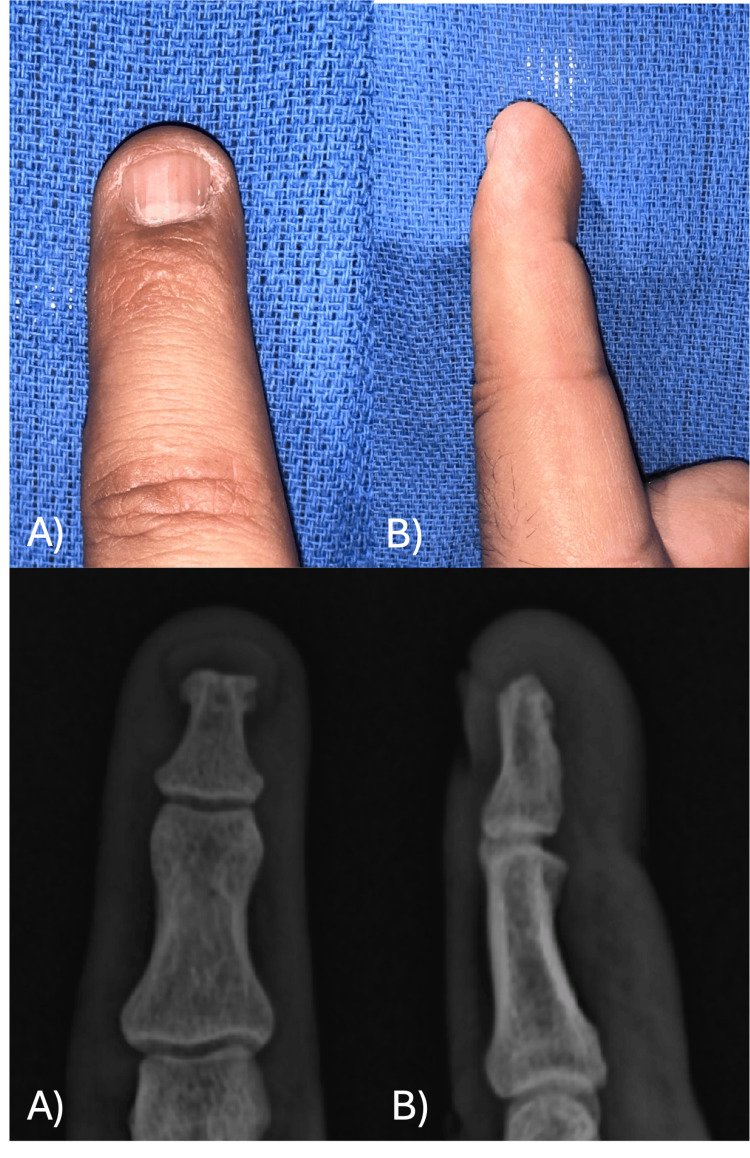
Two-year follow-up images in Case B Very satisfying functional and aesthetic results. (A) Posterior image of index finger. (B) Lateral image of index finger. (C) Posterior X-ray projection. (D) Lateral X-ray projection

The tumor was sent for histopathological study, and it was reported as a glomus tumor fragment measuring 0.8 x 0.4 cm with reparative fibrosis and periostitis (Figure 11). No recurrence of symptoms was reported in two years of follow-up (Figure 12).

## Discussion

Clinical findings described in the literature are variable. Most glomus tumors occur in middle-aged women, while tumors in other anatomical sites are more common in men [1,18]. Our findings were consistent with a higher glomus tumor incidence in women. Consistent with the literature, the average time of disease before diagnosis was 2.9 years, although this may be explained by a couple of outliers who went undiagnosed for 15 years. Furthermore, consistent with the most common location according to the literature, we found that 11 glomus tumors (45.8%) were subungual, representing almost half of all cases.

Glomus tumors were first described over 100 years ago, and there is still innovation in the field. New techniques to decrease the risk of recurrence and improve aesthetic results are currently being developed, such as the newly proposed ultrasound-guided radiofrequency thermocoagulation for glomus tumors [19]. Regarding molecular etiology, over 50% of benign and malignant glomus tumors harbor *MIR143-NOTCH *gene fusions [20], indicating consistent molecular pathogenesis. Further study of immunohistochemical and genetic data is needed to understand glomus tumors and improve their clinical management.

To the best of our knowledge, this is the first case series on glomus tumors of the hand in Mexico, which offers evidence to support that the epidemiology of this geographical region is consistent with published results. However, our study was limited by a small sample size (n = 24) due to the rarity of glomus tumors and short follow-up periods. Further studies with larger sample sizes and longer postoperative follow-up periods are required to strengthen our conclusion.

## Conclusions

Early recognition, individualized case assessment, and selecting an appropriate surgical approach are essential for the effective management of glomus tumors. Evaluating clinical, diagnostic, and intraoperative findings from 24 glomus tumor cases in northern Mexico offers valuable epidemiological insights into regional management. Common features included female predominance, left-hand involvement, especially in the subungual area, and symptoms such as pain, nail bed erythema, localized tenderness, and temperature sensitivity. Most cases were managed using a lateral surgical approach, with bony curettage performed when imaging showed bone involvement. Histopathological confirmation was standard, and recurrence was rare, reinforcing the importance of complete excision.

Ongoing research is needed to reduce recurrence rates further and improve functional and cosmetic outcomes. Novel treatment methods, such as minimally invasive thermal ablation, are under investigation, and genetic studies have begun to identify potential molecular pathways involved in tumor development. Continued examination of histological and genetic markers may lead to more precise and personalized management strategies.

## References

[1] Gombos Z, Zhang PJ (2008). Glomus tumor. Arch Pathol Lab Med.

[2] Mravic M, LaChaud G, Nguyen A, Scott MA, Dry SM, James AW (2015). Clinical and histopathological diagnosis of glomus tumor: an institutional experience of 138 cases. Int J Surg Pathol.

[3] Perţea M, Poroch V, Velenciuc N (2021). Clinical, histopathological and immunohistochemical features of glomus tumor of the nail bed. Rom J Morphol Embryol.

[4] Santoshi JA, Kori VK, Khurana U (2019). Glomus tumor of the fingertips: a frequently missed diagnosis. J Family Med Prim Care.

[5] Grover C, Jayasree P, Kaliyadan F (2021). Clinical and onychoscopic characteristics of subungual glomus tumor: a cross-sectional study. Int J Dermatol.

[6] Abidin MA, Kitta MI, Nong I, Rahmansyah N, Johan MP (2023). Diagnosis and surgical approach in treating glomus tumor distal phalanx left middle finger: a case report. Int J Surg Case Rep.

[7] Samaniego E, Crespo A, Sanz A (2009). Key diagnostic features and treatment of subungual glomus tumor [Article in Spanish]. Actas Dermosifiliogr.

[8] Sun Y, Qi R, Wu Z, Zhang X, Niu J (2024). The clinicopathologic and immunohistochemical features of 60 cutaneous glomus tumor: a retrospective case series study. An Bras Dermatol.

[9] Stewart DR, Sloan JL, Yao L (2010). Diagnosis, management, and complications of glomus tumours of the digits in neurofibromatosis type 1. J Med Genet.

[10] Morey VM, Garg B, Kotwal PP (2016). Glomus tumours of the hand: review of literature. J Clin Orthop Trauma.

[11] Fujioka H, Kokubu T, Akisue T (2009). Treatment of subungual glomus tumor. Kobe J Med Sci.

[12] Huang HP, Tsai MC, Hong KT (2015). Outcome of microscopic excision of a subungual glomus tumor: a 12-year evaluation. Dermatol Surg.

[13] Pandey CR, Singh N, Tamang B (2017). Subungual glomus tumours: is magnetic resonance imaging or ultrasound necessary for diagnosis?. Malays Orthop J.

[14] Netscher DT, Aburto J, Koepplinger M (2012). Subungual glomus tumor. J Hand Surg Am.

[15] Kamble P, Ariwala D, Mohanty SS (2023). The glomus tumor of finger - a case series. J Orthop Case Rep.

[16] Kumar S, Tiwary SK, More R, Kumar P, Khanna AK (2020). Digital glomus tumor: an experience of 57 cases over 20 years. J Family Med Prim Care.

[17] Mathew G, Sohrabi C, Franchi T, Nicola M, Kerwan A, Agha R (2023). Preferred reporting of case series in surgery (PROCESS) 2023 guidelines. Int J Surg.

[18] Dhingra M, Niraula BB, Regmi A, Bansal S, Singh V, Phulware RH (2022). Glomus tumour of hand--a commonly misdiagnosed pathology: a case series. J West Afr Coll Surg.

[19] Wen Y, Lu Z, Li X, Fan X (2023). Ultrasound-guided radiofrequency thermocoagulation for subungual glomus tumor. Asian J Surg.

[20] Mosquera JM, Sboner A, Zhang L (2013). Novel MIR143-NOTCH fusions in benign and malignant glomus tumors. Genes Chromosomes Cancer.

